# An Optimized Method of Metabolite Extraction from Formalin-Fixed Paraffin-Embedded Tissue for GC/MS Analysis

**DOI:** 10.1371/journal.pone.0136902

**Published:** 2015-09-08

**Authors:** Anna Wojakowska, Łukasz Marczak, Karol Jelonek, Krzysztof Polanski, Piotr Widlak, Monika Pietrowska

**Affiliations:** 1 Center for Translational Research and Molecular Biology of Cancer, Maria Sklodowska—Curie Memorial Cancer Center and Institute of Oncology, Gliwice Branch, Wybrzeze Armii Krajowej 15, 44–100, Gliwice, Poland; 2 Institute of Bioorganic Chemistry Polish Academy of Sciences, Noskowskiego 12/14, 61–704 Poznan, Poland; 3 Warwick Systems Biology Centre, University of Warwick, Coventry, CV4 7AL, United Kingdom; Korea University, REPUBLIC OF KOREA

## Abstract

Formalin-fixed paraffin-embedded (FFPE) tissue specimens constitute a highly valuable source of clinical material for retrospective molecular studies. However, metabolomic assessment of such archival material remains still in its infancy. Hence, there is an urgent need for efficient methods enabling extraction and profiling of metabolites present in FFPE tissue specimens. Here we demonstrate the methodology for isolation of primary metabolites from archival tissues; either fresh-frozen, formalin-fixed or formalin-fixed and paraffin-embedded specimens of mouse kidney were analysed and compared in this work. We used gas chromatography followed by mass spectrometry (GC/MS approach) to identify about 80 metabolites (including amino acids, saccharides, carboxylic acids, fatty acids) present in such archive material. Importantly, about 75% of identified compounds were detected in all three types of specimens. Moreover, we observed that fixation with formalin itself (and their duration) did not affect markedly the presence of particular metabolites in tissue-extracted material, yet fixation for 24h could be recommended as a practical standard. Paraffin embedding influenced efficiency of extraction, which resulted in reduced quantities of several compounds. Nevertheless, we proved applicability of FFPE specimens for non-targeted GS/MS-based profiling of tissue metabolome, which is of great importance for feasibility of metabolomics studies using retrospective clinical material.

## Introduction

Formalin fixation followed by paraffin embedding is a standard procedure for stabilization and preservation of tissue samples. The fixation process allows storage of clinical material at ambient temperature over long period of time, while paraffin embedding facilitates preparation of samples for pathological examination. Therefore formalin-fixed paraffin-embedded (FFPE) tissue specimens constitute widely available resources of clinical information, which is essential for retrospective molecular studies. Although fresh-frozen specimens represent a gold standard for genomic, proteomic and metabolomic analysis, their not sufficient availability and expensive storage constitute a serious drawback. In this respect, archival FFPE tissue samples might be a good alternative for fresh-frozen tissue [1–3]. However, process of fixation in formalin results in cross-links between proteins and other biomolecules present in the tissue, owing to reactivity of formaldehyde with side chains of amino acids (e.g. lysine, arginine, tyrosine, asparagine, glutamine) [4]. These crosslinking leads to decreased protein immunoreactivity in immunohistochemical assays. The second limitation in molecular studies is requirement for paraffin removal (which is water insoluble) without damage or loss of targeted compounds. For these reasons, extraction of biomolecules from FFPE material for proteomic and metabolomic studies, particularly for analyses using mass spectrometry techniques, remains a challenging task. Nevertheless, the use of FFPE tissues in retrospective proteomic studies have been widely investigated, and several works described different methods of protein extraction from archival clinical samples [5–7]. Moreover, it was shown that 40–90% of identified proteins overlapped in proteome profiles from fresh-frozen and FFPE tissue samples, depending on the analytical platform [5].

While the number of publications about the use of FFPE tissue in proteomic studies is constantly growing, there is only a few works on metabolomic analysis from this type of clinical material. The first study conducted by Kelly et al. [8] described the targeted LC/MS analysis of polar metabolites extracted from FFPE sarcoma tissue. The authors were able to distinguish malignant from non-malignant tissue samples based on 106 detected metabolites. The same analytical approach was used for the profiling of TCA-cycle-related metabolites in PPGL (pheochromocytomas and paragliomas) tumors, and similar succinate:fumarate ratios were revealed the in fresh-frozen and FFPE tissues [9]. Polar metabolites extracted from FFPE lung and kidney were also analyzed by LC/MS, and distinct metabolic signatures were showed for normal and diseased tissue samples [10]. Moreover, archival FFPE samples were successfully used for detection and quantitation of targeted compounds: 2-hydroxyglutarate [11] and tamoxifen [12], using GC/MS and LC/MS techniques, respectively. However, applicability of FFPE material for GC/MS-based complete metabolite profiling was not validated yet.

In this study we aimed to develop and optimize a method for extraction of primary metabolites from FFPE tissue for GC/MS profiling of general metabolome components. Furthermore, metabolomic profiles obtained from different types of archival tissue material: fresh-frozen, formalin-fixed (then frozen) and FFPE were compared. Obtained results demonstrate the possibility of conducting metabolomic studies using FFPE material, which has a high importance for clinically-oriented research.

## Experimental

### Materials and reagents

Solvents used for extraction and GC-MS analyses were analytical (xylene) or MS grade (methanol, methylene chloride, isopropanol, toluene); derivatization reagents for GC-MS analyses (MSTFA—N-methyl-N-(trimethylsilyl) trifluoroacetamide, O-methylhydroxylamine hydrochloride, pyridine) and alkanes (C10-C36) used as RI (retention index) standards were purchased from Sigma-Aldrich (Poznan, Poland). Deionized water was purified by Milli-Q system (Millipore, Bedford, MA, USA).

### Animals

Five 8-week (±3 days) old male C57Bl/6NCrl mice (22.5g ±0.5g each) were bred in our specific pathogen free (SPF) animal house (Maria Sklodowska-Curie Memorial Cancer Center and Institute of Oncology, Gliwice Branch). Environmental conditions were a temperature of 21°C ±2°C, humidity of 55% ±10%, lighting of 350lx (at bench level) and a 12:12 light:dark cycle. Animals were housed in 207x140x365 mm cages (type IIL, BIOSCAPE, Germany) and given access to mouse food (1324 TPF standard diets, altromin Spezialfutter GmbH & Co. KG, Germany) and water. Environmental enrichment included only bedding (MAXI, LTE-004, ABEDD, Germany). Animals were sacrificed by cervical dislocation. The procedures involving animals and their care were conducted in conformity with the institutional guidelines in compliance with the national and international laws and policies; the study was approved by The Local Ethical Committee for Experiments on Animals, Medical University of Silesia in Katowice, Poland. All sections of this report adhere to the ARRIVE Guidelines for reporting animal research. The complete ARRIVE guidelines checklist is included in S1 Appendix.

### Tissue samples

Pairs of kidneys from each animal were either fixed in formalin (4 mice; one mice for one fixation time) or frozen at -80°C (1 mouse; called fresh-frozen specimen afterwards) immediately after collection.

### Formalin-fixation and paraffin embedding of tissue

**Fig 1:**
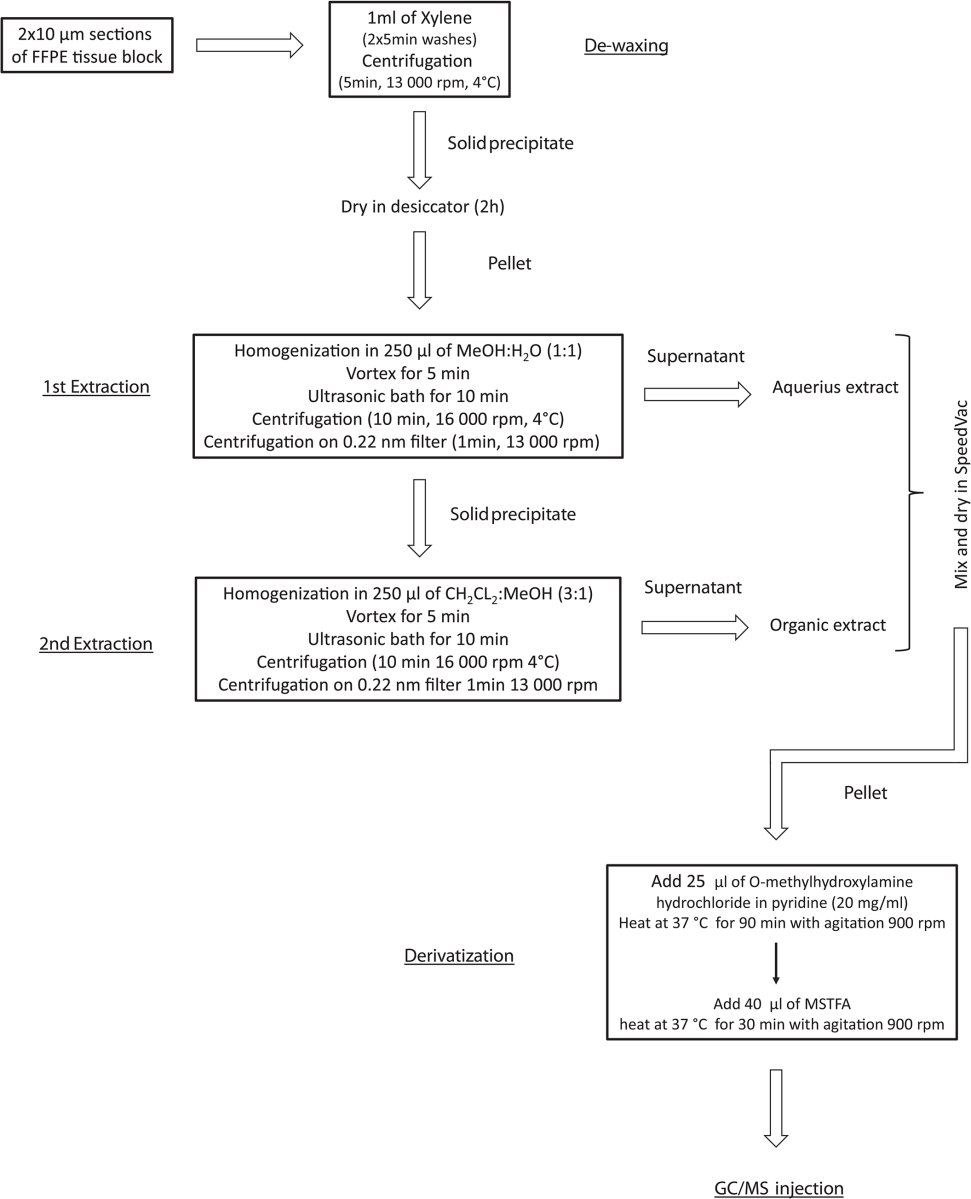
The general workflow of metabolite extraction from formalin-fixed paraffin-embedded tissue blocks for GC/MS analysis.

Kidneys were fixed in buffered formalin (3.7% formaldehyde in 10mM phosphate buffer, pH 7.4) at 4°C for 1, 6, 12, and 24h, respectively, for each of four animals. Then, one kidney from each mouse was directly frozen at -80°C until further analysis (called formalin-fixed specimen afterwards), while another one was prepared for embedding in paraffin (called FFPE specimen afterwards). Kidneys for FFPE preparations were rinsed with 1X PBS at 4°C for 24h (three changes of the buffer, every 2h each, then left overnight), and then dehydrated in alcohol chain: 50% EtOH (1h), 70% EtOH (2h), 80% EtOH (2h), and 96% EtOH (18h). Next, specimens were rinsed twice in 99.8% EtOH for 20min and additionally for 1h, followed by 20min incubation in acetone and washing with xylene (three times, 40min each). Finally, tissues were immersed in 60°C paraffin (twice, 1h each), embedded in a paraffin block and stored at room temperature until further analysis. As a reference we used fresh-frozen kidney (without any further processing step). Each type of tissue sample was processed and analyzed in four technical replicas as shown schematically in Fig 1 and described in details below.

### Extraction of metabolites

Two 10μm-thick sections of each tissue preparation were placed in 1.5mL cup-locked tubes. Deparaffinization of FFPE samples was performed by two rounds of washing with 1mL of xylene followed by centrifugation for 5 minutes at 13,000rpm in 4°C; solid pellet was dried in desiccator for 2h. Dried deparaffinized FFPE samples, as well as formalin-fixed and fresh-frozen samples were extracted as described below (see flowchart in Fig 1). Samples were homogenized in 250μL mixture of MeOH:H_2_O (1:1 v/v), vortexed for 5 min and placed in an ultrasonic bath for 10min, and the resulting mixture was centrifuged for 10min at 16,000rpm in 4°C. The supernatant was transferred to centrifugal filter units (Millipore PVDF 0.22μm), and then centrifuged for 1min at 13,000rpm to generate polar fraction (1^st^ extraction). The pellet was suspended in 250μL of mixture of Ch_2_Cl_2_:MeOH (3:1 v/v) and processed as described above to generate non-polar fraction (2^nd^ extraction). Both polar and non-polar fractions were combined, transferred to a new tube and evaporated in a SpeedVac concentrator. Dried extract was then derivatized with 25μL of methoxyamine hydrochloride in pyridine (20mg/mL) at 37°C for 90min with agitation. The second step of derivatization was performed by adding 40μL of MSTFA and incubation at 37°C for 30min with agitation. Samples were subjected to GC/MS analysis directly after derivatization. Each sample was prepared in four repetitions. Compounds were considered “identified” when they met the identification criteria established by GC software used (LECO ChromaTOF), namely: identity score higher than 700, Mass Threshold higher than 10 and matched retention index.

### GC/MS analysis

The analysis was performed using Agilent 7890A gas chromatograph (Agilent Technologies) connected to Pegasus 4D GCxGC-TOFMS mass spectrometer (Leco). A DB-5 bonded-phase fused-silica capillary column of 30m length, 0.25mm inner diameter and 0.25μm film thickness (J&W Scientific Co.,USA) was used for separation. The GC oven temperature program was as follows: 2min at 70°C, raised by 8°C/min to 300°C and held for 16min at 300°C. The total time of GC analysis was 46.75min. Helium was used as the carrier gas at a flow rate of 1mL/min. One microliter of each derivatized sample was injected in a splitless mode. The initial PTV (Programmed Temperature Vaporization) injector temperature was 20°C for 0.1min and after that time raised by 600°C/min to 350°C. The septum purge flow rate was 3mL/min and the purge was turned on after 60s. The transfer line and ion source temperatures were set to 250°C. In-source fragmentation was performed with 70eV energy. Mass spectra were recorded in the mass range 35–650 *m/z*.

### Analysis of spectra

Data acquisition, automatic peak detection, mass spectrum deconvolution, retention index calculation and library search were done by Leco ChromaTOF-GC software (v4.51.6.0). To eliminate retention time (Rt) shift and to determine the retention indexes (RI) for each compound, the alkane series mixture (C-10 to C-36) was injected into the GC/MS system. The metabolites were automatically identified by library search (Replib, Mainlib, Fiehn library) with similarity index (SI) above 700 and RI +/- 10. All known artifact peaks including alkanes, plasticizers, column bleed, MSTFA artifact and reagent peaks, were not considered in the final results. To obtain accurate peak areas for the deconvoluted components, unique quantification masses for each component were specified and the samples were reprocessed. The obtained metabolite data were normalized relative to the sum of chromatographic peak area (using the TIC approach) in each sample before statistical analyses.

### Statistical analysis

The pairwise comparisons were performed using either the t-test, Welch test or U-Mann-Whitney test, depending on normality distribution and homoscedasticity of analyzed data. The multiple group analyses were performed using either the Kruskal-Wallis test or ANOVA test, depending on normality distribution and homoscedasticity of analyzed data. The normality distribution was assessed using the Kolmogorov-Smirnov test, while homoscedasticity was tested using the F test of equality of variances. The Benjamini-Hochberg assessment of False Discovery Rate (FDR) was applied for multiple testing correction in each case.

## Results and Discussion

### Comparison of metabolite extraction and identification in different types of tissue preparations

The general workflow of metabolite extraction from FFPE tissue blocks for GC/MS analysis, which flowchart is showed in Fig 1, includes deparaffinization by xylene treatment followed by extraction with mixtures of water and methanol, then methylene chloride and methanol. This two-phase extraction protocol was developed based on method described for fresh-frozen tissue by Denkert et al. [13]. The same extraction protocol was applied for tissues specimens that did not require deparaffinization, i.e. fresh-frozen and formalin-fixed ones. The described procedure was successfully applied for profiling of metabolites present in FFPE tissue sections using GC/MS technique–resulting metabolomics profiles were comparable with profiles obtained using fresh-frozen or formalin-fixed (not followed by paraffin-embedding) material, which is described in more detail below. To the best of our knowledge, this is the first GC/MS-based protocol demonstrating metabolome profiling of FFPE tissue. It should be emphasized that paraffin removal is the first and most critical step in processing of FFPE tissue specimen. A previous study describing an LC/MS-based approach for metabolite profiling of FFPE specimens used removal of paraffin by melting in hot methanol [8]. However, our results revealed that deparaffinization method described in that paper cannot be adopted for subsequent GC/MS analysis (very low yield of metabolite identification was observed with this method). Moreover, treatment with xylene (or other xylene substitute like hexane) is the most common approach to remove paraffin. Sahm et al. [11] previously described a method for detection 2-hydroxyglutarate from FFPE glioma specimens using GC/MS, where deparaffinization was performed just by adding xylene. Here we proved that FFPE material deparaffinized by a xylene treatment can be also used for general non-targeted metabolome profiling. Fixation of tissue in formalin results in cross-links between proteins and nucleic acids owing to preferentially reactivity of formaldehyde with side chains of amino acids [14]. However, in contrast to proteomic studies, the metabolomic approach does not require antigen retrieval in order to cross-link reversion, hence this step could be omitted in presented procedure for processing of FFPE specimens.

In this work we aimed to compare GC/MS profiles of metabolites extracted from FFPE specimen and from material that did not require deparaffinization (i.e., either fresh-frozen or just formalin-fixed). Mouse kidney was selected as an experimental model because of its availability and the fact that metabolomic profile of mouse tissue was thoroughly investigated in previous studies [15], hence we were able to verify obtained data. Small molecules were tentatively identified by comparison of the experimental mass spectra with GC/MS library databases. To achieve a better accuracy in library matches, retention indices were used (compounds with a similarity index below 700 were not included in the results). This approach allowed for identification of about 80 metabolites in general, including amino acids, carbohydrates, carboxylic acids, fatty acids, esters of fatty acids, nucleosides, sterols, sugar alcohols and others. Complete list of detected metabolites is presented in Table 1.

**Table 1:** Metabolites detected by GC/MS in different preparations of mouse kidney–comparison between different types of tissue preparations.

Compound class	Metabolite name	FFPE/FrFr		FF/FrFr	
		ratio	p-value	ratio	p-value
Aminoacids	Alanine	0.30	0.01	1.16	0.64
	Aspartic acid	0.08	0.01	0.78	0.38
	Glutamic acid	n.d.^a^	-	2.02	0.21
	Glycine	0.13	0.03	0.26	0.01
	Lysine	0.24	0.003	1.36	0.73
	Ornithine	n.d.^a^	-	0.24	0.002
	Serine	0.19	0.19	2.29	0.08
	Threonine	n.d.^a^	-	0.95	0.78
	Tyrosine	n.d.^a^	-	1.67	0.23
	Thiazolidine-4-carboxylic acid	n.d.^a^	-	0.80	0.50
	Valine	0.25	0.01	1.03	0.93
	5-Oxoproline	0.18	0.01	0.83	0.51
Saccharides	Erythrose	0.81	0.17	0.85	0.58
	Galactose	n.d.^a^	-	0.35	0.01
	Glucose	0.11	0.01	4.07	0.10
	Lyxose	2.67	0.05	1.29	0.58
	Tagatose	n.d.^a^	-	1.90	0.21
	Xylose	n.d.^a,c^	-	n.d.^a,c^	-
Sugar alcohols	Glycerol	0.94	0.73	1.71	0.24
	Myo-inositol	0.08	0.02	8.89	0.01
	Ribitol	1.19	0.29	1.03	0.93
	Scyllo-inositol	0.39	0.01	2.92	0.06
Carboxilic acids	Benzoic acid	1.91	0.12	1.65	0.28
	Citric acid	0.30	0.002	0.83	0.46
	Glyoxylic acid	0.21	0.0002	1.15	0.82
	Glyceric acid	1.49	0.27	0.83	0.40
	Glycolic acid	n.d.^a^	-	1.11	0.59
	Lactic acid	0.34	0.01	0.60	0.12
	Malic acid	n.d.^a^	-	26.00	0.002
	Pyruvic acid	n.d.^a,c^	-	n.d.^a,c^	-
	Succinic acid	0.63	0.16	7.86	0.03
	Thiodiacetic acid	0.92	0.45	0.90	0.56
	Valeric acid	1.30	0.05	1.19	0.41
Fatty acids	Arachidonic acid	0.23	0.004	1.74	0.20
	Arachidic acid	1.70	0.10	1.17	0.59
	Caproic acid	1.04	0.63	1.21	0.40
	Cervonic acid	n.d.^a^	-	1.03	0.87
	Linoleic acid	2.53	0.01	1.42	0.25
	Linolenic acid	1.40	0.21	0.80	0.49
	Margaric acid	1.28	0.16	1.05	0.87
	Myristic acid	1.42	0.12	0.97	0.87
	Nonadecanoic acid	1.40	0.17	1.16	0.59
	Oleic acid	0.70	0.22	0.66	0.64
	Palmitelaidic acid	0.37	0.03	1.34	0.25
	Palmitic acid	1.15	0.39	1.13	0.67
	Pelargonic acid	0.76	0.38	0.76	0.25
	Pentadecanoic acid	1.31	0.13	1.06	0.83
	Ricinoleic acid	0.67	0.53	1.04	0.83
	Stearic acid	1.37	0.13	1.08	0.80
	10-Undecenoic acid	1.61	0.17	0.57	0.21
Fatty acids esters	Monopalmitoylglycerol	2.10	0.10	1.16	0.78
	Dodecanoic acid 1-methylethyl ester	0.93	0.60	1.45	0.55
	Eicosanoic acid propyl ester	0.75	0.44	0.96	0.92
	Myristic acid propyl ester	1.50	0.22	1.21	0.64
	Heptadecanoic acid glycerine-(1)-monoester	0.74	0.70	1.29	0.58
	Hexadecanoic acid methyl ester	0.35	0.003	0.67	0.25
	Hexadecanoic acid propyl ester	1.61	0.25	1.35	0.58
	Nonadecanoic acid glycerine-(1)-monoester	0.67	0.19	1.24	0.58
	9-Octadecenoic acid propyl ester	2.16	0.05	1.25	0.59
	9-Octadecenoic acid methyl ester	1.49	0.06	0.88	0.59
	9,12,15-Octadecatrienoic acid propyl ester	0.97	0.79	0.94	0.67
	Pentadecanoic acid glycerine-(1)-monoester	2.20	0.07	1.36	0.55
	Glyceryl stearate	0.71	0.53	1.34	0.58
Nucleosides	Adenosine	n.d.^a^	-	2.02	0.49
	Inosine	n.d.^a^	-	0.54	0.25
Sterols	Cholesterol	n.d.^a^	-	0.51	0.38
	4-Methyl-cholesta-8,24-dien-3-ol	2.04	0.12	0.39	0.19
Others metabolites	Ethosuximide (drug)	n.d.^b,c^	-	n.d.^b,c^	-
	Gluconic acid	0.28	0.0009	2.33	0.08
	Glucuronic acid	n.d.^a,c^	-	n.d.^a,c^	-
	Gluconic acid lactone	0.45	0.05	1.18	0.55
	Glycerol 3-phosphate	0.12	0.0004	3.15	0.06
	Myo-inositol phosphate	0.27	0.04	0.79	0.58
	2-Phosphoglyceric acid	0.30	0.01	1.09	0.71
	2-Deoxy-erythrose-phosphate	n.d.^a^	-	1.55	0.49
	2-Deoxy-erythro-pentonic acid	n.d.^a^	-	0.45	0.19
	2-Aminoethyl phosphoric acid	0.08	0.001	0.62	0.21
	Urea	0.45	0.10	0.85	0.46

Shown are differences (fold-changes) in relative abundances of each metabolite between FFPE or formalin-fixed (FF) tissue specimens and fresh-frozen (FrFr) reference tissue, followed by statistical significance of the difference (p-value estimated by the t-test). Tissue specimens were fixed with formalin for 24h. Metabolites not detected (n.d.) in FFPE, FF or FrFr specimens are marked with ^a, b^ and ^c^, respectively.

**Fig 2:**
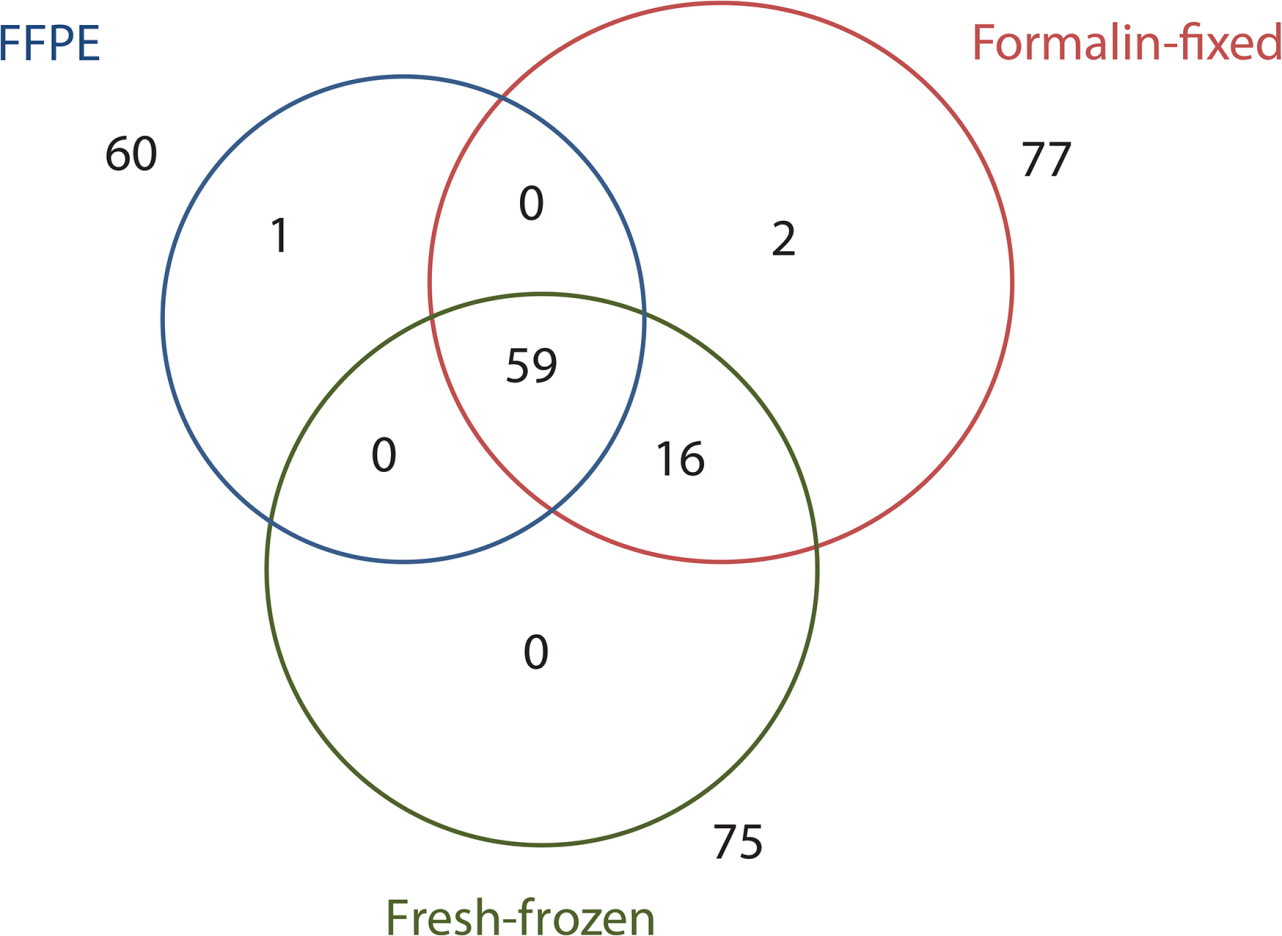
Venn diagram showing numbers of identified compounds detected in extracts from fresh-frozen, formalin-fixed and FFPE specimens of mouse kidney (samples were fixed with formalin for 24h).

Fig 2 presents the Venn diagram showing the overlap in the number of metabolites identified in material extracted from three types of compared tissue samples (24h of fixation in formalin was used at this point). In general, 75, 77 and 60 compounds was detected in fresh-frozen, formalin-fixed and FFPE specimens, respectively. About 75% of identified metabolites was detected in all three types of specimens, while 96% of identified metabolites was detected in both types of specimens not subjected to deparaffinization procedure (i.e. fresh-frozen and formalin-fixed ones). For the most classes of metabolites, except for the nucleosides not detected in FFPE material, at least 50% of the identified molecules were present in all three types of tissue specimens. All fatty acids esters and sugar alcohols, 94% of fatty acids and 70% of carboxylic acids were detected in all three types of tissue samples. The largest variation in levels of identified molecules was detected among amino acids, saccharides and carboxylic acids when statistical significance of differences in abundance of particular molecules between different types of samples was assessed (Table 1 and S1 Table). Nevertheless, we concluded that significant number of metabolites that belong to different classes of compound overlapped in FFPE, formalin-fixed and fresh-frozen tissue specimens.

**Fig 3:**
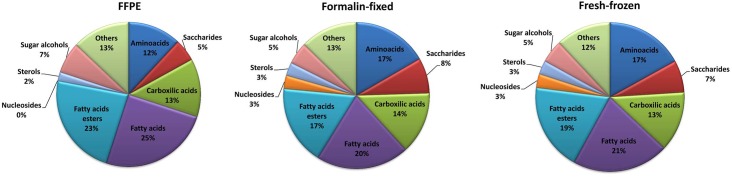
Contribution of different classes of metabolites detected in extracts from fresh-frozen, formalin-fixed or FFPE specimens of mouse kidney (samples were fixed with formalin for 24h).

Fig 3 shows the distribution of different classes of small molecules detected in three types of tissue samples. The largest and most abundant group of molecules were fatty acids and their esters, constituting about 40% of identified molecular species in all types of preparations. It has been generally assumed that deparaffinization with xylene leads to leaching out lipids from the tissue, therefore lipidomic analysis of dewaxed samples may be unreliable. However, recent study Hughes et al. [16] shows that washing of the tissue in xylene or hexane for 5–10 minutes leaches out only unbound tissue lipids, while most of membrane lipids remain present in deparaffinazed FFPE tissue. Here we confirmed that these classes of small molecules could be reliably analysed in archive FFPE material. Unexpectedly, the highest qualitative and quantitative differences between tissue deparaffinized with xylene and tissues processed without this treatment were observed for polar water-soluble compounds–nucleosides, amino acids, and saccharides. With exception of fatty acids, fatty acids esters and sugar alcohols, for other classes of small metabolites there were several compounds missing in the FFPE material (Table 1 and S1 Table). Furthermore, the abundances of several metabolites were lower in FFPE samples compared to fresh-frozen or formalin-fixed samples. Significant differences were observed in the content of amino acids, whose the lowest levels were detected in FFPE specimen. The same trend was observed in case of most carboxylic acids, saccharides and sugar alcohols. On the other hand, in case of several lipids, including fatty acids and their esters, the highest abundances were detected in extracts from FFPE specimen (Table 1 and S1 Table). Hence, one could hypothesize that fatty acids identified in FFPE tissue samples were solvent-resistant part of the membrane protein lipid complex [16]. Moreover, relative abundances of several metabolites were lower in fresh-frozen samples than in formalin-fixed samples, yet only in case of six compounds (glycine, ornithine, galactose, myo-inositol, malic and succinic acid) the differences were statistically significant (this phenomenon could result from the TIC approach used for sample normalization). One should assume that all steps of tissue processing (like formalin fixation, deparaffinization, and extraction with organic solvents) could result in degradation and/or attrition of particular molecular species, which was previously observed in transcriptomic and proteomic studies using FFPE specimens [6,17,18]. Recent studies provide evidence that certain water-soluble molecules (e.g. amino acids, carbohydrates, lipids, phosphates and proteins) leach away from tissue specimen into the aqueous fixative medium during formalin fixation of the sections [19]. Moreover, Kelly et al. [8] reported markedly lower number of metabolites identified by LC/MS in extracts from FFPE tissue when compared to other LC/MS-based studies using fresh-frozen material. However, because of a lack of GC/MS-based metabolomics studies using FFPE tissue material, efficacy of extraction methods used in present study cannot be compared directly with other reports using similar experimental design. Nevertheless, we showed here that FFPE tissue specimen can be used for general non-targeted metabolome profiling by GS/MS. However, conclusions based on direct comparison of such data with data obtained using fresh-frozen or just formalin-fixed material should be apparently avoided.

### Influence of time of formalin fixation on metabolite recovery

Tissue samples resected surgically are routinely fixed in formalin for further evaluation by pathologist. Although different times of fixation are in use, fixation for 18–24h is the most frequent setting in clinical practice. Several proteomic studies demonstrated that fixation time affects the protein recovery and variability in peptide and protein identification [20,21]. Hackett et al. [19] reported that formalin fixation leaches out water-soluble molecules (including metabolites) from tissue sections, and hypothesized that prolonged time of fixation allow accumulation of mobile biomolecules into aqueous fixative medium. However, there is a lack of validated reports about the influence of fixation time on results of metabolomic studies. To address this issue we compared profiles of metabolites extracted from tissue fixed in formalin for 1, 6, 12 and 24h, in both FFPE and formalin-fixed type of samples. Molecular species identified in all such tissue preparations are listed in S2 Table. The number of overlapped compounds detected in both types of samples fixed in formalin for different times and fresh-frozen reference is presented in the Venn diagrams showed in S1 Fig. In general, we did not observed large differences in numbers of molecular species identified in tissue specimens fixed for different time with buffered solution of formalin. We noted that about 64%, 68%, and 70% of identified molecules were detected in all three types of specimens, when tissues were fixed with formalin for 1, 6 and 12h, respectively (compared to 75% of common molecules for 24h of fixation). Furthermore, no particular class of metabolites was depleted when shorter times of fixation were compared with 24-hour “standard” (see S2 Table). Moreover, we did not observe large differences in abundance of particular molecules between tissue specimens fixed for different time with formalin (S2 Table), in neither FFPE nor formalin-fixed samples. These data apparently indicate that increased duration of fixation in formalin has no negative impact on the compatibility of FFPE and formalin-fixed tissue material with GS/MS-based metabolite profiling. To the best of our knowledge this is the first reported study addressing the impact of formalin-fixation time on results of metabolomic analysis, hence any comparison with the results of other studies is not available. Moreover, published data on the influence of fixation time on proteomic analyses of FFPE specimen are inconclusive—some works suggest that increased duration of fixation has a negative impact on the suitability of FFPE samples for proteomic analysis [21], while others stay in opposite [20]. Hence, considering the fact that one-day fixation with formalin is a frequent practice in clinical settings, and that the largest number of metabolites common for fresh-frozen and formalin-fixed specimens were observed in tissue fixed for 24h, we could recommend this time of formalin fixation as a standard for metabolomic studies.

## Conclusions

Formalin fixation followed by paraffin embedding is a gold standard of sample preparation widely applied in pathology laboratories, while fresh-frozen samples are not available in majority of clinical studies. Hence, there is an urgent need establishing a standardized, efficient method of extraction of metabolites from FFPE tissue. Here we present a method for successful extraction and GC/MS-based analysis of metabolites from FFPE tissue. Our results clearly demonstrate that the number of identified metabolites is only slightly impacted by formalin fixation itself. Paraffin embedding has more influence on extraction efficiency, and results in affected levels of some compounds when compared to fresh-frozen specimens. Nevertheless, we have shown applicability of FFPE specimens for non-targeted GS/MS-based profiling of tissue metabolome, which is of great importance for feasibility of retrospective studies for clinical metabolomics.

## Supporting Information

S1 Appendix The ARRIVE Guidelines Checklist for Animal Research: Reporting In Vivo Experiments. (PDF)

S1 Fig Venn diagrams comparing numbers of identified compounds detected in extracts from fresh-frozen, formalin-fixed and FFPE specimens of mouse kidney—samples were fixed with formalin for 1, 6 and 12h. (EPS)

S1 Table Abundances of metabolites detected by GC/MS in different preparations of mouse kidney. (DOCX)

S2 Table Metabolites detected by GC/MS in mouse kidney fixed with formalin for different time. (DOCX)
