# Supplementary material for: An Optimized Method of Metabolite Extraction from Formalin-Fixed Paraffin-Embedded Tissue for GC/MS Analysis
Source: PLoS One. 2015 Sep 8;10(9):e0136902. doi: 10.1371/journal.pone.0136902 (PMC4562636; doi:10.1371/journal.pone.0136902)
Supplement: S1 Table — (DOCX) [file pone.0136902.s003.docx]

Table S1. Abundances of metabolites detected by GC/MS in different preparations of mouse kidney.

| **Compound class** | **Metabolite name** | **Amounts of metabolites** | | | | | | **Difference** (p-value) |
| --- | --- | --- | --- | --- | --- | --- | --- | --- |
|  |  | **FFPE** | | **Formalin-fixed** | | **Fresh-frozen** | |  |
|  |  | **mean value** [a.u.] | **RSD** [%] | **mean value** [a.u.] | **RSD** [%] | **mean value** [a.u.] | **RSD** [%] |  |
| Aminoacids | Alanine | 3.01 | 41.9 | 11.50 | 30.4 | 9.89 | 15.1 | 0.01 |
|  | Aspartic acid | 0.34 | 97.1 | 3.41 | 34.7 | 4.40 | 11.0 | 0.008 |
|  | Glutamic acid | n.d. | - | 4.09 | 46.4 | 2.02 | 14.2 | - |
|  | Glycine | 5.25 | 83.6 | 10.67 | 32.8 | 41.49 | 13.9 | 0.0002 |
|  | Lysine | 1.85 | 28.2 | 10.64 | 116.3 | 7.82 | 16.7 | 0.36* |
|  | Ornithine | n.d. | - | 0.13 | 68.5 | 0.54 | 14.7 | - |
|  | Serine | 0.71 | 90.6 | 3.83 | 35.6 | 1.67 | 15.9 | 0.03 |
|  | Threonine | n.d | - | 0.38 | 47.2 | 0.40 | 22.7 | - |
|  | Tyrosine | n.d. | - | 0.90 | 34.3 | 0.54 | 17.9 | - |
|  | Thiazolidine-4-carboxylic acid | n.d. | - | 1.29 | 35.1 | 1.62 | 20.9 | - |
|  | Valine | 0.63 | 80.8 | 3.39 | 25.7 | 3.28 | 13.9 | 0.01 |
|  | 5-Oxoproline | 2.77 | 52.8 | 12.81 | 31.6 | 15.48 | 17.0 | 0.008 |
| Saccharides | Erythrose | 0.39 | 11.2 | 0.41 | 44.6 | 0.48 | 11.7 | 0.67 |
|  | Galactose | n.d. | - | 6.52 | 24.8 | 18.39 | 15.0 | - |
|  | Glucose | 0.25 | 40.6 | 9.49 | 53.4 | 2.33 | 42.2 | 0.003 |
|  | Lyxose | 0.56 | 27.4 | 0.27 | 37.6 | 0.21 | 49.7 | 0.03 |
|  | Tagatose | n.d. | - | 0.19 | 32.1 | 0.10 | 35.0 | - |
|  | Xylose | n.d. | - | 11.44 | 34.4 | n.d. | - | - |
| Sugar alcohols | Glycerol | 1149.07 | 28.3 | 2089.92 | 38.1 | 1221.07 | 16.3 | 0.12 |
|  | Myo-inositol | 1.64 | 90.7 | 173.91 | 47.5 | 19.56 | 14.2 | 0.12* |
|  | Ribitol | 1.41 | 14.7 | 1.22 | 26.3 | 1.18 | 19.2 | 0.54 |
|  | Scyllo-inositol | 1.83 | 19.3 | 13.80 | 40.6 | 4.72 | 16.5 | 0.12* |
| Carboxilic acids | Benzoic acid | 4.38 | 39.3 | 3.78 | 42.8 | 2.29 | 12.5 | 0.24 |
|  | Citric acid | 0.36 | 20.7 | 1.00 | 22.0 | 1.20 | 17.2 | 0.005 |
|  | Glyoxylic acid | 0.27 | 67.4 | 1.48 | 42.1 | 1.29 | 13.1 | 0.05 |
|  | Glyceric acid | 0.61 | 37.9 | 0.34 | 24.0 | 0.41 | 11.2 | 0.14 |
|  | Glycolic acid | n.d. | - | 0.82 | 17.8 | 0.74 | 13.5 | 0.02 |
|  | Lactic acid | 53.93 | 27.3 | 94.02 | 26.5 | 157.24 | 14.4 | 0.005 |
|  | Malic acid | n.d. | - | 1.30 | 40.8 | 0.05 | 67.7 | - |
|  | Pyruvic acid | n.d. | - | 0.21 | 68.8 | n.d. | - | - |
|  | Succinic acid | 1.50 | 34.5 | 18.71 | 75.5 | 2.38 | 22.9 | 0.12* |
|  | Thiodiacetic acid | 17.40 | 2.6 | 16.97 | 16.1 | 18.86 | 13.1 | 0.56 |
|  | Valeric acid | 1.12 | 3.8 | 1.02 | 16.2 | 0.86 | 15.2 | 0.11 |
| Fatty acids | Arachidonic acid | 0.31 | 8.5 | 2.33 | 24.0 | 1.34 | 31.1 | 0.005 |
|  | Arachidic acid | 3.74 | 11.2 | 2.57 | 20.4 | 2.20 | 38.8 | 0.06 |
|  | Caproic acid | 2.72 | 4.1 | 3.16 | 18.8 | 2.62 | 11.8 | 0.27 |
|  | Cervonic acid | n.d. | - | 1.59 | 24.6 | 1.54 | 32.7 | - |
|  | Linoleic acid | 1.85 | 12.6 | 1.04 | 21.2 | 0.73 | 22.7 | 0.005 |
|  | Linolenic acid | 0.21 | 27.5 | 0.12 | 38.4 | 0.15 | 15.9 | 0.13 |
|  | Margaric acid | 7.66 | 8.3 | 6.31 | 26.5 | 6.00 | 21.0 | 0.31 |
|  | Myristic acid | 20.97 | 18.7 | 14.42 | 24.7 | 14.81 | 18.5 | 0.11 |
|  | Nonadecanoic acid | 1.02 | 7.6 | 0.85 | 23.8 | 0.73 | 33.7 | 0.24 |
|  | Oleic acid | 0.61 | 15.2 | 0.57 | 73.0 | 0.87 | 32.7 | 0.11 |
|  | Palmitelaidic acid | 0.10 | 76.1 | 0.37 | 21.4 | 0.27 | 21.7 | 0.02 |
|  | Palmitic acid | 290.18 | 14.0 | 286.61 | 27.1 | 252.82 | 17.3 | 0.49 |
|  | Pelargonic acid | 0.48 | 53.3 | 0.48 | 23.4 | 0.63 | 6.8 | 0.51 |
|  | Pentadecanoic acid | 3.19 | 12.9 | 2.58 | 23.4 | 2.44 | 17.1 | 0.21 |
|  | Ricinoleic acid | 0.18 | 27.6 | 0.28 | 32.5 | 0.27 | 43.3 | 0.40 |
|  | Stearic acid | 546.65 | 7.4 | 430.42 | 27.4 | 398.57 | 25.5 | 0.20 |
|  | 10-Undecenoic acid | 0.79 | 29.3 | 0.28 | 30.2 | 0.49 | 28.7 | 0.02 |
| Fatty acids esters | Monopalmitoylglycerol | 17.35 | 24.0 | 9.56 | 48.8 | 8.25 | 52.2 | 0.09 |
|  | Dodecanoic acid 1-methylethyl ester | 1.82 | 4.9 | 2.82 | 46.3 | 1.95 | 15.2 | 0.60* |
|  | Eicosanoic acid propyl ester | 0.38 | 33.0 | 0.49 | 35.9 | 0.51 | 32.2 | 0.56 |
|  | Myristic acid propyl ester | 0.21 | 22.8 | 0.17 | 28.8 | 0.14 | 39.8 | 0.35 |
|  | Heptadecanoic acid glycerine-(1)-monoester | 1.11 | 32.1 | 1.92 | 34.4 | 1.49 | 55.3 | 0.37 |
|  | Hexadecanoic acid methyl ester | 4.52 | 19.5 | 8.70 | 43.8 | 12.94 | 14.8 | 0.02 |
|  | Hexadecanoic acid propyl ester | 93.10 | 28.4 | 77.80 | 41.5 | 57.69 | 57.7 | 0.83 |
|  | Nonadecanoic acid glycerine-(1)-monoester | 0.14 | 28.6 | 0.26 | 26.7 | 0.21 | 25.3 | 0.11 |
|  | 9-Octadecenoic acid propyl ester | 2.81 | 15.4 | 1.63 | 39.1 | 1.30 | 45.0 | 0.04 |
|  | 9-Octadecenoic acid methyl ester | 1.46 | 10.4 | 0.86 | 29.2 | 0.98 | 20.4 | 0.03 |
|  | 9,12,15-Octadecatrienoic acid propyl ester | 18.58 | 1.5 | 17.91 | 16.9 | 19.13 | 11.23 | 0.80 |
|  | Pentadecanoic acid glycerine-(1)-monoester | 0.55 | 20.3 | 0.34 | 38.5 | 0.25 | 48.7 | 0.15 |
|  | Glyceryl stearate | 76.59 | 40.0 | 144.10 | 35.1 | 107.83 | 53.4 | 0.30 |
| Nucleosides | Adenosine | n.d. | - | 1.03 | 50.6 | 0.51 | 51.0 | - |
|  | Inosine | n.d. | - | 0.98 | 59.6 | 1.81 | 34.2 | - |
| Sterols | Cholesterol | n.d. | - | 10.03 | 53.8 | 19.71 | 45.5 | - |
|  | 4-Methyl-cholesta-8,24-dien-3-ol | 2.96 | 27.8 | 0.56 | 38.5 | 1.45 | 46.0 | 0.01 |
| Others metabolites | Ethosuximide (drug) | 0.13 | 70.6 | n.d. | - | n.d. | - | - |
|  | Gluconic acid | 1.68 | 13.6 | 13.91 | 38.4 | 5.98 | 13.7 | 0.12* |
|  | Glucuronic acid | n.d. | - | 0.20 | 62.3 | n.d. | - | - |
|  | Gluconic acid lactone | 0.20 | 74.3 | 0.52 | 21.7 | 0.44 | 13.4 | 0.06 |
|  | Glycerol 3-phosphate | 0.48 | 9.9 | 12.98 | 39.2 | 4.12 | 23.2 | 0.12* |
|  | Myo-inositol phosphate | 0.31 | 51.3 | 0.91 | 22.2 | 1.15 | 33.7 | 0.02 |
|  | 2-Phosphoglyceric acid | 0.30 | 33.7 | 1.10 | 20.1 | 1.01 | 23.6 | 0.008 |
|  | 2-Deoxy-erythrose-phosphate | n.d. | - | 1.38 | 37.7 | 0.89 | 38.7 | - |
|  | 2-Deoxy-erythro-pentonic acid | n.d. | - | 4.24 | 34.8 | 9.48 | 43.6 | - |
|  | 2-Aminoethyl phosphoric acid | 0.09 | 79.8 | 0.70 | 35.6 | 1.13 | 19.2 | 0.008 |
|  | Urea | 1.53 | 57.4 | 2.92 | 23.4 | 3.42 | 12.6 | 0.04 |

Relative quantification is based on TIC normalization and showed in arbitrary units (a.u.); mean values and relative standard deviation (RSD given as a percent) from four replicas are presented. Significance of differences among all three types of specimens was estimated by the ANOVA or Kruscal-Wallis* test depending on data normality. Tissue specimens were fixed with formalin for 24 hours.
