# Supplementary material for: An Optimized Method of Metabolite Extraction from Formalin-Fixed Paraffin-Embedded Tissue for GC/MS Analysis
Source: PLoS One. 2015 Sep 8;10(9):e0136902. doi: 10.1371/journal.pone.0136902 (PMC4562636; doi:10.1371/journal.pone.0136902)
Supplement: S2 Table — (DOCX) [file pone.0136902.s004.docx]

Table S2. Metabolites detected by GC/MS in mouse kidney fixed with formalin for different time

| **Compound class** | **Metabolite name** | **1h-FFPE/FrFr** | | **6h-FFPE/FrFr** | | **12h-FFPE/FrFr** | | **24h-FFPE/FrFr** | | **1h-FF/FrFr** | | **6h-FF/FrFr** | | **12h-FF/FrFr** | | **24h-FF/FrFr** | |
| --- | --- | --- | --- | --- | --- | --- | --- | --- | --- | --- | --- | --- | --- | --- | --- | --- | --- |
|  |  | ratio | p-value | ratio | p-value | ratio | p-value | ratio | p-value | ratio | p-value | ratio | p-value | ratio | p-value | ratio | p-value |
| Aminoacids | Alanine | 0.24 | <0.01 | n.d.^a^ | - | 1.15 | 0.86 | 0.30 | 0.01 | 1.40 | 0.99 | 0.55 | 0.26 | n.d.^b^ | - | 1.16 | 0.64 |
|  | Aspartic acid | n.d.^a^ | - | 0.03 | <0.01 | 0.33 | 0.09 | 0.08 | 0.01 | 1.15 | 0.93 | 0.52 | 0.44 | 0.15 | 0.07 | 0.78 | 0.38 |
|  | Glutamic acid | n.d.^a^ | - | n.d.^a^ | - | 0.10 | <0.01 | 0.00^a^ | - | 3.18 | 0.90* | 0.77 | 0.74 | 0.15 | 0.04 | 2.02 | 0.21 |
|  | Glycine | 0.03 | <0.01 | 0.07 | <0.01 | 0.41 | 0.09 | 0.13 | 0.03 | 0.91 | 0.90* | 0.16 | 0.03 | 0.07 | 0.01 | 0.26 | 0.01 |
|  | Lysine | 0.48 | 0.08 | 2.34 | 0.01 | 1.68 | 0.32 | 0.24 | <0.01 | 1.42 | 0.96* | 0.97 | 0.88 | 1.15 | 0.80 | 1.36 | 0.73 |
|  | Ornithine | n.d.^a^ | - | n.d.^a^ | - | n.d.^a^ | - | n.d.^a^ | - | 1.36 | 0.96* | n.d.^b^ | - | n.d.^b^ | - | 0.24 | <0.01 |
|  | Phenylalanine | n.d.^a,c^ | - | n.d.^a,c^ | - | n.d.^a,c^ | - | n.d.^a,c^ | - | n.d.^c^ | - | n.d.^b,c^ | - | n.d.^b,c^ | - | n.d.^b,c^ | - |
|  | Serine | n.d.^a^ | - | 0.24 | <0.01 | 5.51 | 0.40 | 0.19 | 0.19 | 3.23 | 0.90* | 1.16 | 0.87 | 0.58 | 0.33 | 2.29 | 0.08 |
|  | Threonine | n.d.^a^ | - | n.d.^a^ | - | n.d.^a^ | - | n.d.^a^ | - | 2.23 | 0.88 | 0.60 | 0.59 | n.d.^b^ | - | 0.95 | 0.78 |
|  | Tyrosine | n.d.^a^ | - | n.d.^a^ | - | 1.22 | 0.87 | n.d.^a^ | - | 3.43 | 0.90* | 0.90 | 0.78 | n.d.^b^ | - | 1.67 | 0.23 |
|  | Thiazolidine-4-carboxylic acid | n.d.^a^ | - | n.d.^a^ | - | n.d.^a^ | - | n.d.^a^ | - | 3.08 | 0.90* | n.d.^b^ | - | n.d.^b^ | - | 0.80 | 0.50 |
|  | Valine | 0.19 | <0.01 | 0.22 | <0.01 | 1.29 | 0.93 | 0.25 | 0.01 | 2.05 | 0.90* | 1.02 | 0.97 | n.d.^b^ | - | 1.03 | 0.93 |
|  | 5-Oxoproline | 0.13 | 0.01 | 0.16 | 0.01 | 0.45 | 0.12 | 0.18 | 0.01 | 1.30 | 0.97* | 0.65 | 0.59 | 0.36 | 0.06 | 0.83 | 0.51 |
| Saccharides | Erythrose | n.d.^a^ | - | n.d.^a^ | - | n.d.^a^ | - | 0.81 | 0.17 | 0.94 | 0.90* | 1.10 | 0.86 | 1.21 | 0.65 | 0.85 | 0.58 |
|  | Galactose | n.d.^a^ | - | n.d.^a^ | - | n.d.^a^ | - | n.d.^a^ | - | 0.47 | 0.90* | 0.37 | 0.19 | 0.17 | 0.02 | 0.35 | 0.01 |
|  | Glucose | 0.12 | 0.01 | 0.14 | 0.01 | 0.08 | <0.01 | 0.11 | 0.01 | 4.38 | 0.96* | 1.11 | 0.97 | 0.35 | 0.21 | 4.07 | 0.10 |
|  | Lyxose | 2.99 | 0.02 | 2.84 | 0.05 | 2.68 | 0.05 | 2.67 | 0.05 | 2.09 | 0.88 | 1.71 | 0.44 | 2.73 | 0.26 | 1.29 | 0.58 |
|  | Tagatose | n.d.^a^ | - | n.d.^a^ | - | n.d.^a^ | - | n.d.^a^ | - | 1.32 | 0.99 | n.d.^b^ | - | 1.92 | 0.55 | 1.90 | 0.21 |
|  | Xylose | n.d.^a,c^ | - | n.d.^a,c^ | - | n.d.^a,c^ | - | n.d.^a,c^ | - | n.d.^c^ | - | n.d.^c^ | - | n.d.^bc^ | - | n.d.^c^ | - |
| Sugar alcohols | Glycerol | 0.69 | 0.03 | 0.82 | 0.34 | 0.81 | 0.19 | 0.94 | 0.73 | 1.13 | 0.96 | 1.10 | 0.81 | 0.92 | 0.73 | 1.71 | 0.24 |
|  | Myo-inositol | 0.06 | <0.01 | 0.11 | <0.01 | 0.14 | <0.01 | 0.08 | 0.02 | 3.22 | 0.90* | 1.98 | 0.59 | 0.72 | 0.42 | 8.89 | 0.01 |
|  | Ribitol | 1.79 | 0.08 | 1.06 | 0.70 | 0.97 | 0.88 | 1.19 | 0.29 | 0.92 | 0.90* | 1.11 | 0.78 | 1.36 | 0.59 | 1.03 | 0.93 |
|  | Scyllo-inositol | 1.07 | 0.80 | 0.75 | 0.34 | 0.54 | 0.03 | 0.39 | 0.01 | 2.74 | 0.90* | 1.38 | 0.78 | 1.03 | 0.87 | 2.92 | 0.06 |
| Carboxilic acids | Benzoic acid | 0.93 | 0.56 | 2.36 | <0.01 | 1.87 | 0.05 | 1.91 | 0.12 | 1.58 | 1.00* | 1.05 | 0.91 | 1.11 | 0.86 | 1.65 | 0.28 |
|  | Citric acid | 0.16 | <0.01 | 0.34 | 0.06 | 0.91 | 0.61 | 0.30 | <0.01 | 1.23 | 0.96* | 0.96 | 0.91 | 0.93 | 0.76 | 0.83 | 0.46 |
|  | Glyoxylic acid | n.d.^a^ | - | n.d.^a^ | - | n.d.^a^ | - | 0.21 | <0.01 | 1.22 | 0.96* | 0.62 | 0.44 | n.d.^b^ | - | 1.15 | 0.82 |
|  | Glyceric acid | 1.28 | 0.24 | 1.12 | 0.44 | 1.16 | 0.29 | 1.49 | 0.27 | 0.95 | 0.90* | 0.96 | 0.86 | 0.90 | 0.68 | 0.83 | 0.40 |
|  | Glycolic acid | n.d.^a^ | - | n.d.^a^ | - | n.d.^a^ | - | n.d.^a^ | - | n.d.^b^ | - | n.d.^b^ | - | n.d.^b^ | - | 1.11 | 0.59 |
|  | Lactic acid | 0.40 | <0.01 | 0.35 | <0.01 | 0.40 | <0.01 | 0.34 | 0.01 | 0.58 | 0.90* | 0.39 | 0.03 | 0.59 | 0.40 | 0.60 | 0.12 |
|  | Malic acid | n.d.^a^ | - | n.d.^a^ | - | n.d.^a^ | - | n.d.^a^ | - | n.d.^b^ | - | n.d.^b^ | - | n.d.^b^ | - | 26.00 | <0.01 |
|  | Pyruvic acid | n.d.^c^ | - | n.d.^a,c^ | - | n.d.^a,c^ | - | n.d.^a,c^ | - | n.d.^b,c^ | - | n.d.^b,c^ | - | n.d.^c^ | - | n.d.^c^ | - |
|  | Succinic acid | 1.01 | 0.90 | 0.91 | 0.65 | 0.69 | 0.12 | 0.63 | 0.16 | 5.69 | 0.90* | 2.06 | 0.45 | 1.95 | 0.32 | 7.86 | 0.03 |
|  | Thiodiacetic acid | 1.36 | 0.03 | 1.22 | 0.44 | 1.09 | 0.51 | 0.92 | 0.45 | 0.76 | 0.90* | 1.14 | 0.74 | 1.44 | 0.40 | 0.90 | 0.56 |
|  | Valeric acid | 2.39 | <0.01 | 1.88 | 0.01 | 1.61 | 0.04 | 1.30 | 0.05 | 0.90 | 0.90* | 0.79 | 0.26 | 1.97 | 0.20 | 1.19 | 0.41 |
| Fatty acids | Arachidonic acid | n.d.^a^ | - | n.d.^a^ | - | 0.28 | 0.01 | 0.23 | <0.01 | 1.21 | 0.96 | 0.98 | 0.97 | 2.13 | 0.26 | 1.74 | 0.20 |
|  | Arachidic acid | 2.41 | 0.04 | 2.01 | 0.11 | 1.79 | 0.11 | 1.70 | 0.10 | 1.87 | 0.88 | 1.44 | 0.50 | 2.66 | 0.25 | 1.17 | 0.59 |
|  | Caproic acid | 1.53 | 0.02 | 1.29 | 0.17 | 1.17 | 0.25 | 1.04 | 0.63 | 0.95 | 0.90* | 1.06 | 0.87 | 1.91 | 0.23 | 1.21 | 0.40 |
|  | Cervonic acid | n.d.^a^ | - | n.d.^a^ | - | n.d.^a^ | - | n.d.^a^ | - | 0.68 | 0.88 | 0.66 | 0.59 | 1.61 | 0.55 | 1.03 | 0.87 |
|  | Linoleic acid | 7.25 | <0.01 | 4.71 | <0.01 | 3.83 | 0.01 | 2.53 | 0.01 | 2.18 | 0.88 | 1.78 | 0.19 | 2.77 | 0.13 | 1.42 | 0.25 |
|  | Linolenic acid | n.d.^a^ | - | n.d.^a^ | - | 1.33 | 0.80 | 1.40 | 0.21 | 1.15 | 0.99* | 0.88 | 0.78 | 1.32 | 0.52 | 0.80 | 0.49 |
|  | Margaric acid | 1.19 | 0.23 | 1.29 | 0.52 | 1.34 | 0.27 | 1.28 | 0.16 | 1.25 | 0.98 | 1.09 | 0.81 | 1.77 | 0.33 | 1.05 | 0.87 |
|  | Myristic acid | 1.35 | 0.07 | 1.31 | 0.35 | 1.30 | 0.23 | 1.42 | 0.12 | 1.17 | 0.99* | 1.06 | 0.87 | 1.61 | 0.37 | 0.97 | 0.87 |
|  | Nonadecanoic acid | 1.57 | 0.10 | 1.50 | 0.31 | 1.49 | 0.21 | 1.40 | 0.17 | 1.43 | 0.93 | 1.17 | 0.76 | 2.39 | 0.24 | 1.16 | 0.59 |
|  | Oleic acid | 1.40 | 0.67 | 1.03 | 0.99 | 0.74 | 0.38 | 0.70 | 0.22 | 0.84 | 0.88 | 0.79 | 0.73 | 1.68 | 0.33 | 0.66 | 0.64 |
|  | Palmitelaidic acid | 2.39 | 0.36 | 0.84 | 0.44 | 0.61 | 0.09 | 0.37 | 0.03 | 0.85 | 0.90* | 0.79 | 0.59 | 2.08 | 0.24 | 1.34 | 0.25 |
|  | Palmitic acid | 1.18 | 0.29 | 1.14 | 0.70 | 1.16 | 0.51 | 1.15 | 0.39 | 1.11 | 0.96* | 1.04 | 0.91 | 1.57 | 0.41 | 1.13 | 0.67 |
|  | Pelargonic acid | 1.00 | 0.94 | 0.68 | 0.09 | 0.48 | 0.01 | 0.76 | 0.38 | 0.76 | 0.90* | 0.79 | 0.26 | 1.72 | 0.26 | 0.76 | 0.25 |
|  | Pentadecanoic acid | 1.85 | 0.07 | 1.53 | 0.18 | n.d.^a^ | - | 1.31 | 0.13 | 1.21 | 0.99* | 1.07 | 0.87 | 1.66 | 0.39 | 1.06 | 0.83 |
|  | Ricinoleic acid | 0.72 | 0.58 | 1.01 | 0.94 | n.d.^a^ | - | 0.67 | 0.53 | 1.07 | 0.95 | 0.76 | 0.81 | 1.54 | 0.40 | 1.04 | 0.83 |
|  | Stearic acid | 1.34 | 0.13 | 1.43 | 0.35 | 1.41 | 0.21 | 1.37 | 0.13 | 1.40 | 0.95 | 1.18 | 0.74 | 1.85 | 0.33 | 1.08 | 0.80 |
|  | 10-Undecenoic acid | 0.51 | 0.25 | 1.68 | 0.13 | 0.95 | 0.85 | 1.61 | 0.17 | 0.37 | 0.88 | 1.00 | 0.94 | 0.77 | 0.53 | 0.57 | 0.21 |
| Fatty acids esters | Monopalmitoylglycerol | 1.86 | 0.09 | 2.26 | 0.11 | 2.28 | 0.09 | 2.10 | 0.10 | 2.14 | 0.88 | 1.49 | 0.59 | 2.11 | 0.39 | 1.16 | 0.78 |
|  | Dodecanoic acid 1-methylethyl ester | 3.23 | <0.01 | 1.12 | 0.84 | 0.95 | 0.78 | 0.93 | 0.60 | 0.84 | 0.90* | 1.01 | 0.97 | 3.45 | 0.04 | 1.45 | 0.55 |
|  | Eicosanoic acid propyl ester | 1.93 | 0.20 | 1.34 | 0.55 | 0.58 | 0.32 | 0.75 | 0.44 | 0.91 | 0.88 | 0.81 | 0.78 | 1.71 | 0.33 | 0.96 | 0.92 |
|  | Myristic acid propyl ester | 1.96 | 0.03 | 1.40 | 0.31 | 1.51 | 0.16 | 1.50 | 0.22 | 1.20 | 0.99 | 1.26 | 0.76 | 2.12 | 0.35 | 1.21 | 0.64 |
|  | Heptadecanoic acid glycerine-(1)-monoester | 1.46 | 0.26 | 0.98 | 0.87 | 0.82 | 0.85 | 0.74 | 0.70 | 1.20 | 0.96 | 0.96 | 0.91 | 2.03 | 0.52 | 1.29 | 0.58 |
|  | Hexadecanoic acid methyl ester | 0.33 | <0.01 | 0.38 | 0.01 | 0.26 | <0.01 | 0.35 | <0.01 | 0.56 | 0.90* | 0.92 | 0.78 | 0.76 | 0.43 | 0.67 | 0.25 |
|  | Hexadecanoic acid propyl ester | 2.36 | 0.06 | 1.93 | 0.17 | 2.01 | 0.19 | 1.61 | 0.25 | 1.76 | 0.88 | 1.28 | 0.74 | 2.00 | 0.39 | 1.35 | 0.58 |
|  | Nonadecanoic acid glycerine-(1)-monoester | 0.90 | 0.67 | 0.97 | 0.92 | n.d.^a^ | - | 0.67 | 0.19 | 0.99 | 0.90* | 0.95 | 0.87 | 1.65 | 0.43 | 1.24 | 0.58 |
|  | 9-Octadecenoic acid propyl ester | 2.70 | 0.03 | 3.13 | 0.06 | 2.19 | 0.06 | 2.16 | 0.05 | 2.64 | 0.88 | 1.71 | 0.45 | 2.94 | 0.20 | 1.25 | 0.59 |
|  | 9-Octadecenoic acid methyl ester | 1.24 | 0.46 | 1.47 | 0.34 | 1.43 | 0.16 | 1.49 | 0.06 | 1.18 | 0.98 | 0.97 | 0.91 | 1.60 | 0.42 | 0.88 | 0.59 |
|  | 9,12,15-Octadecatrienoic acid propyl ester | 1.36 | 0.02 | 1.19 | 0.34 | 1.10 | 0.45 | 0.97 | 0.79 | 0.95 | 0.90* | 1.12 | 0.76 | 1.42 | 0.48 | 0.94 | 0.67 |
|  | Pentadecanoic acid glycerine-(1)-monoester | 3.44 | 0.01 | 2.82 | 0.03 | 2.46 | 0.06 | 2.20 | 0.07 | 1.62 | 0.88 | 1.65 | 0.53 | 2.87 | 0.23 | 1.36 | 0.55 |
|  | Glyceryl stearate | 1.10 | 0.77 | 0.92 | 0.99 | 0.67 | 0.48 | 0.71 | 0.53 | 1.25 | 0.94 | 1.01 | 0.91 | 1.74 | 0.59 | 1.34 | 0.58 |
| Nucleosides | Adenosine | n.d.^a^ | - | n.d.^a^ | - | n.d.^a^ | - | n.d.^a^ | - | 1.23 | 0.88 | 0.94 | 0.91 | 0.87 | 0.80 | 2.02 | 0.49 |
|  | Inosine | n.d.^a^ | - | n.d.^a^ | - | n.d.^a^ | - | n.d.^a^ | - | n.d.^b^ | - | 0.36 | 0.13 | 0.75 | 0.54 | 0.54 | 0.25 |
| Sterols | Cholesterol | 2.02 | 0.73 | 0.18 | 0.03 | n.d.^a^ | - | n.d.^a^ | - | 0.63 | 0.88 | 0.73 | 0.78 | 0.78 | 0.75 | 0.51 | 0.38 |
|  | 4-Methyl-cholesta-8,24-dien-3-ol | 0.35 | 0.03 | 2.02 | 0.11 | 1.08 | 0.95 | 2.04 | 0.12 | 0.42 | 0.88 | 0.78 | 0.81 | 0.46 | 0.29 | 0.39 | 0.19 |
| Others metabolites | Ethosuximide (drug) | n.d.^c^ | - | n.d.^c^ | - | n.d.^c^ | - | n.d.^c^ | - | n.d.^b,c^ | - | n.d.^b,c^ | - | n.d.^b,c^ | - | n.d.^b,c^ | - |
|  | Gluconic acid | 0.36 | 0.01 | 0.43 | 0.04 | 1.24 | 0.73 | 0.28 | <0.01 | 3.01 | 0.90* | 0.95 | 0.83 | 4.65 | 0.24 | 2.33 | 0.08 |
|  | Glucuronic acid | n.d.^a,c^ | - | n.d.^a,c^ | - | n.d.^a,c^ | - | n.d.^a,c^ | - | n.d.^b,c^ | - | n.d.^b,c^ | - | n.d.^b,c^ | - | n.d.^c^ | - |
|  | Gluconic acid lactone | 1.09 | 0.61 | 0.99 | 0.90 | 0.71 | 0.09 | 0.45 | 0.05 | 1.40 | 0.99* | 1.18 | 0.76 | 1.95 | 0.29 | 1.18 | 0.55 |
|  | Glycerol 3-phosphate | 0.16 | <0.01 | 0.15 | <0.01 | 0.13 | <0.01 | 0.12 | <0.01 | 0.96 | 0.90* | 0.82 | 0.76 | 0.80 | 0.55 | 3.15 | 0.06 |
|  | Myo-inositol phosphate | 0.09 | <0.01 | 0.19 | 0.01 | 0.24 | 0.01 | 0.27 | 0.04 | 0.48 | 0.88 | 0.45 | 0.19 | 0.74 | 0.55 | 0.79 | 0.58 |
|  | 2-Phosphoglyceric acid | 0.30 | <0.01 | 0.27 | 0.01 | 0.29 | <0.01 | 0.30 | 0.01 | 0.67 | 0.90* | 0.47 | 0.10 | 0.97 | 0.84 | 1.09 | 0.71 |
|  | 2-Deoxy-erythrose-phosphate | n.d.^a^ | - | n.d.^a^ | - | n.d.^a^ | - | n.d.^a^ | - | 0.38 | 0.88 | 0.43 | 0.34 | 0.56 | 0.38 | 1.55 | 0.49 |
|  | 2-Deoxy-erythro-pentonic acid | n.d.^a^ | - | n.d.^a^ | - | n.d.^a^ | - | n.d.^a^ | - | n.d.^b^ | - | n.d.^b^ | - | 0.70 | 0.47 | 0.45 | 0.19 |
|  | 2-Aminoethyl phosphoric acid | n.d.^a^ | - | n.d.^a^ | - | 0.08 | <0.01 | 0.08 | <0.01 | 1.49 | 0.99* | 0.58 | 0.50 | 0.14 | 0.01 | 0.62 | 0.21 |
|  | Urea | n.d.^a^ | - | 0.33 | <0.01 | 0.59 | 0.09 | 0.45 | 0.10 | 4.04 | 0.90* | 1.25 | 0.55 | 0.68 | 0.43 | 0.85 | 0.46 |

Shown are differences (fold-changes) in relative abundances of each metabolite between FFPE or formalin-fixed (FF) tissue specimens and fresh-frozen (FrFr) reference tissue, followed by statistical significance of the difference (p-value estimated by the t-test or Welch test*, depending on normality of data). Tissue specimens were fixed with formalin for 1, 6, 12 or 24 hours. Metabolites not detected (n.d.) in FFPE, FF or FrFr specimens are marked with ^a, b^ and ^c^ , respectively.
